# Thermal Benefits From White Variegation of *Silybum marianum* Leaves

**DOI:** 10.3389/fpls.2019.00688

**Published:** 2019-05-24

**Authors:** Oren Shelef, Liron Summerfield, Simcha Lev-Yadun, Santiago Villamarin-Cortez, Roy Sadeh, Ittai Herrmann, Shimon Rachmilevitch

**Affiliations:** ^1^Department of Natural Resources, Institute of Plant Sciences, Agricultural Research Organization, Rishon LeZion, Israel; ^2^French Associates Institute for Agriculture and Biotechnology of Drylands, The Jacob Blaustein Institutes for Desert Research, Ben-Gurion University of the Negev, Beersheba, Israel; ^3^Department of Biology and Environment, Faculty of Natural Sciences, University of Haifa–Oranim, Tivon, Israel; ^4^Biology Department, University of Nevada, Reno, Reno, NV, United States; ^5^The Robert H. Smith Institute of Plant Sciences and Genetics in Agriculture, The Robert H. Smith Faculty of Agriculture, Food and Environment, The Hebrew University of Jerusalem, Rehovot, Israel

**Keywords:** patch, *Silybum marianum*, leaf color, thermoregulation, IRGA, plant physiology

## Abstract

Leaves of the spiny winter annual *Silybum marianum* express white patches (variegation) that can cover significant surface areas, the outcome of air spaces formed between the epidermis and the green chlorenchyma. We asked: (1) what characterizes the white patches in *S. marianum* and what differs them from green patches? (2) Do white patches differ from green patches in photosynthetic efficiency under lower temperatures? We predicted that the air spaces in white patches have physiological benefits, elevating photosynthetic rates under low temperatures. To test our hypotheses we used both a variegated wild type and entirely green mutants. We grew the plants under moderate temperatures (20°C/10°C d/n) and compared them to plants grown under lower temperatures (15°C/5°C d/n). The developed plants were exposed to different temperatures for 1 h and their photosynthetic activity was measured. In addition, we compared in green vs. white patches, the reflectance spectra, patch structure, chlorophyll and dehydrin content, stomatal structure, plant growth, and leaf temperature. White patches were not significantly different from green patches in their biochemistry and photosynthesis. However, under lower temperatures, variegated wild-type leaves were significantly warmer than all-green mutants – possible explanations for that are discussed These findings support our hypothesis, that white variegation of *S. marianum* leaves has a physiological role, elevating leaf temperature during cold winter days.

## Introduction

Most live leaves in land plants contain chlorophyll and therefore appear to the human eye as green. Evolution drives plants to produce considerable amounts of green tissues in order to pursue sufficient photosynthesis. This selection pressure is especially pronounced in annuals, which are active during a limited and often very short season. However, in many vascular plants a prominent portion of the leaves is not green. Leaves of the Mediterranean spiny winter annual rosette plant *Silybum marianum* (Asteraceae) express white patches (variegation) that can cover significant areas of its leaves. We asked, is there a potential physiological advantage of that white variegation. Plant scientists suggested several non-exclusive explanations to non-green plant coloration patterns (Jiang et al., 2004). These possible mechanisms can be sorted to three different groups: (1) Chlorophyll deficiency (Aluru et al., 2001; Sheue et al., 2012); (2) defense from enemies including by aposematic coloration, undermining the camouflage of herbivorous insects, mimicry of dead or infested plants, masquerade and camouflage (Lev-Yadun et al., 2004; Lev-Yadun, 2016, 2017; Niu et al., 2018); (3) white patterns as secondary byproducts of advantageous physiological structures such as adaptations for improved water or gas transport (Fooshee and Henny, 1990), or an unknown role (Tsukaya et al., 2004); (4) white patterns provide other physiological adaptations including mitigation of UV radiation (Roelfsema et al., 2006) and thermoregulation. White variegation in *S. marianum* as defense from herbivores by various methods was studied by Lev-Yadun (2003; 2009; 2016) suggested that white variegation in dozens of species with spiny leaves are related to protection against herbivores via visual aposematism, showing a positive correlation between white patchiness and spine number and size. White variegation presumably also mimic pest tunnels (e.g., Smith, 1986; Lev-Yadun, 2016), and signal both invertebrate and mammalian herbivores to avoid eating the already infested leaves (e.g., Soltau et al., 2009). Delayed greening is also related to anti-herbivory defense in tropical ecosystems (Kursar and Coley, 1992). Another interesting hypothesis is related to physiological explanations. This hypothesis suggests that bundle sheath extensions (BSEs) have a role in water transport between the mesophyll and the vascular tissue through the epidermis (McClendon, 1992; Buckley et al., 2011). Accumulating evidence suggest that BSEs play a role in mitigating high radiation in accordance with photosynthetic activity and leaf structure, and as a function of nutrition and water status (Barbosa et al., 2018). Unlike leaf variegation, BSEs are apparent only with transmitted light. Nevertheless – this is an example of a structural leaf feature that provides physiological plasticity to the plant. Xiao et al. (2016) have extensively studied the role of anatomical features in light absorptance. If variegation is structural, it may explain a possible physiological role.

A common type of variance in coloration is defined as variegation, and is a result of either pigment-expression-related heterogeneity, or differences in leaf structure (Sheue et al., 2012). Variegation in tropical leaves has been hypothesized to create a cellular lens structure, as an adaptation for poor light conditions. Two different types of structural variegation are known (Tsukaya et al., 2004) – the “air-space” is a variation in palisade-cell development, in which the palisade cells exhibit rounded shape with air spaces that produce pale grayish-green appearance, as can be found in *Begonia* spp. The second type, i.e., “epidermis” type of variegation, was described in several species, e.g., *Oxalis mariana*, where epidermal attachment to the underlying cell layers varies across a leaf, and sub-epidermal air spaces appear as white or whitish due to their stronger light reflectance (Hara, 1957). Konoplyova et al. (2008) showed that epidermal variegation does not result in loss of photosynthetic capacity. According to this concept variegation is an adaptive morphology leading to non-uniform photosynthesis (Terashima, 1992). Nevertheless, Brodersen and Vogelmann (2007) showed that lens shaped epidermal cells were not advantageous in capturing of indirect (diffuse) light, suggesting that other traits, such as leaf thickness, are more important. In *S. marianum*, the white leaf variegation is the outcome of the formation of sub-epidermal air spaces (Lev-Yadun, 2017).

Several researchers linked thermal advantage to the micro-environment formed by white patches (Hüner et al., 2012). They hypothesized that under cold conditions, bright patches form a microenvironment that alleviate the impact of temperatures to allow better conditions for photosynthesis. Avoidance of freezing and of low temperatures in plants is a widely reported phenomenon (Weiser, 1970; Pearce, 2001; Sakai and Larcher, 2012), but despite the recognition that anatomical structures such as leaf hardness contribute to this adaptation, the role of plant coloration as effecting warming was not studied extensively except for the translucent bracts of several *Rheum* species growing in very high elevations in the Himalaya (Omori and Ohba, 1996; Omori et al., 2000; Tsukaya, 2002; Song et al., 2013). Plants are exposed to various temperatures seasonally or diurnally, hence thermoregulation of these changes has a potentially enormous effect on physiological plant activity in general and specifically on cell photostasis (Hüner et al., 2012) and on photosynthetic rate (Berry and Bjorkman, 1980). Structural modifications in higher plants were reported in alpine and nival regions as an adaptation to low temperatures and to a short vegetation period (Lütz and Engel, 2007).

We hypothesized that by alleviating leaf temperatures, white patches in *S. marianum* leaves provide a microenvironment that promotes photosynthesis rate under the low morning temperatures prevailing in the mildly low winter temperatures that characterize the growth season of this Mediterranean annual. A well-known parallel adaptation under these conditions is diaheliotropism - sun-tracking by leaf movement (Koller, 2000). We are not aware of any study that tested this hypothesis in *S. marianum* or in other annuals with similar white patches (i.e., *Notobasis syriaca*). Our main questions were: (1) what is the nature of white patches in *S. marianum*? What differentiate them from green patches? Specifically we studied the physiological performance of white vs. green patches. (2) Do white patches specifically exhibit more efficient photosynthesis under lower temperatures? We predicted that chlorophyll content of white and green patches is not significantly different and that the morphological difference between the two color appearances allows white patches to provide improved microenvironment. We predicted that this microenvironment effect alleviates low-temperature stress and provides thermic advantage under low temperatures with sufficient photosynthetic active radiation. To test this hypothesis we compared green and white patches of *S. marianum*. We studied morphological traits (stomata abundance and cuticle shape), optic attributes (transmittance, reflectance, and absorptance) biochemistry (chlorophyll, total carotene, and dehydrin content) and physiological performance (photosynthetic activity in different conditions of temperature and light). We also compared the wild type *S. marianum* expressing the white patches to mutants with green leaves.

## Materials and Methods

### Plant Collection and Growth

*Silybum marianum* (L.) Gaertn. (Asteraceae) is a spiny rosette winter annual native to the Mediterranean basin, where it is widespread as a common ruderal weed. Historical records report that the seeds of milk thistle (*S. marianum*) were long used to treat liver related deficiencies (Křen and Walterova, 2005), and recent literature suggested that it does have a real medicinal impact (Karkanis et al., 2011), as well as a nutritional value (Vaknin et al., 2008). The branched canopy is 40–300 cm high, (Montemurro et al., 2007). The basal leaves are alternate, large and glabrous with spiny margins. Leaves of well-developed plants are commonly 50–60 cm long and 20–30 cm wide, and have typical white veins (Gresta et al., 2007), although leaves of very large rosettes may be more than 90 cm long (Lev-Yadun, 2003). Its main growth period is in the early winter, when day temperatures are usually relatively low (5–15°C) and water is available (Supplementary Material S1). The seeds are 5–8 mm long, formed at the center of a spiny inflorescence head about 5 cm in diameter, and typically pink in color, although white inflorescence heads are common in various populations (Keasar et al., 2016). Seeds for the plants grown for our study were collected from a wild population in Israel. For comparative studies, we used a wild occurring mutant that appears to have all green leaves, with no white variegation at all. 13 populations of such non-variegated mutants were found in various localities in Israel (Lev-Yadun, 2017). All-green mutant plants growing in a mixed population containing both all-green mutants and variegated wild type were marked by flags at the growing season. Seeds were collected from those marked all-green mutant plants growing in the wild at Nes Ziyyona (about 10 km south of Tel-Aviv) (e.g., Lev-Yadun, 2017).

### Experimental Design

In order to study the properties and function of white patches and to compare them to green patches under different temperature conditions, we used the following experimental setup. We compared three different types of *S. marianum* leaf patches: (1) an entirely green leaf in mutant plants; (2) white patches in wild type variegated leaves; (3) green patches in wild type variegated leaves. We sowed seeds in a greenhouse, one type per 10 liter pot, to establish plants for 3 weeks. The plants were grown in a potting soil. The two plant type seedlings (wild-type and all-green mutant) were then transferred and grown for 15 days in two controlled growth chambers (Percival, Perry, IA). The schedule light regime was 11.5/12.5 d/n with daily intensity of 600 PPFD, and with relative humidity (RH) kept on 70%. Plants were grown individually in 4 liter pots. For statistical power, 15 individuals per variety where used as replicates in each growth chamber with the following temperature levels. Normal temperature simulated an average Mediterranean winter conditions (20°C/10°C d/n); cold temperature (15°C/5°C d/n). All measurements were done on these six patches and temperature types: three patch types (all-green mutant, both green and white patches in wild-type plants), two temperature growth conditions (normal/cold). All experiments took place at December 2012–February 2013.

### Hyperspectral Data Collection With Integrating Sphere

To explore the leaf radiation balance, a FieldSpec PRO (Analytical Spectral Devices, Inc., [ASD], Longmont, CO, United States) was used with an Integrating Sphere I-800-12 (LI-COR Biosciences; Lincoln, NE, United States) as fore optics. This system allows reflectance and transmittance data collection that are used to calculate the leaf absorptance (Gitelson and Merzlyak, 1997; Herrmann et al., 2017). Measurements were acquired from 15 plants including 8 all-green mutants and 7 wild types with a total of 17 samples (few mixed patches were obtained from the same plant). Hyperspectral was obtained from four different patch types: (1) green patches on all-green mutants (eight samples); (2) white patches on wild type leaves (three samples); (3) green mixed where mixed patches spots on wild type, dominated by the green color (three samples); and (4) white mixed where mixed patches spots on wild type plants, dominated by white color (three samples). The mixed type dominancy was estimated by pictures obtained during data collection (% white patch). The spectral range presented (400–1,700 nm) can be divided to three main regions, based on their main influence on the spectral data: visible (VIS; 400–700 nm) mainly affected by pigments content, near infra-red (NIR; 700–1,300 nm) influenced mostly by the leaf internal structure, and shortwave infrared (SWIR; 1,300–1,700 nm) mainly influenced by water absorptance (Curran, 1989; Herrmann et al., 2010). The reflectance and transmittance were measured and the absorptance was calculated. The spectral data was averaged per patch type and measuring method.

### Patch Structure

Five variegated wild type leaves and five leaves of all-green mutant plants were sampled. In the variegated wild type leaves, the samples included half of a white sector and half of a green sector. The samples were immediately fixed in a mixture of 3:1 ethanol and glacial acetic acid overnight at room temperature. After fixation, samples were washed in water three times for 15 min each, dehydrated overnight in a series of ethanol solutions (25, 50, 75, 96, and 100%), and embedded in paraffin. Serial cross sections, 5–10 μm thick, were prepared with a rotary microtome (American Optical model 820, Spencer), from the sampled leaf segments, stained with Safranin and fast green, and mounted with Permount (Thermo Fisher Scientific, Cat. No. SP15-100). Slides were examined under bright field with a Leitz Dialux 20 microscope equipped with a Nikon F3 camera, at magnifications of X16 to X400.

### Stomata Structure

Stomata structure was estimated from epidermal impressions following Falik et al. (2011, 2012) and Sachs et al. (1993). The abaxial and adaxial surfaces of mature leaves were copied using a fresh mixture of vinyl polysiloxane dental impression material (Elite HD +, Badia Polesine, Rovigo, Italy). The resulting hardened imprints were further copied with clear nail polish, which resulted in transparent preparations suitable for microscopic examination. For statistical repetitions, we used five imprints, from five different plants. Measurements of stomata size and density were carried out using AxioVision software (Carl Zeiss MicroImaging, Thornwood, NY, United States) on digital images of the nail-polish preparations.

### Phytochemical Content (Chlorophyll, Total Carotene and Dehydrin)

Chlorophyll-*a*, chlorophyll-*b* and carotenes were extracted in acetone (80%) and estimated from mature leaves, following the standard method of Arnon (1949). These pigments have a pivoting role in utilizing sun radiation. Hence, to study differences between patches, we wanted to estimate the biochemical characteristics of each patch type. Total carotene was quantified according to the formulas of Lichtenthaler and Wellburn (1985). Dehydrins (DHNs) are an important group of proteins, providing drought stress tolerance (Close and Bray, 1993; Yu et al., 2018) among other functions to the plant. Dehydrin was extracted following methods described by Nylander et al. (2001). The dehydrin content was evaluated by western blot analysis. Proteins were extracted in 50 mM Tris–HCl, 250 mM sucrose, 5 mM EDTA, 10 mM 2-mercaptoethanol pH 7.2, containing protease inhibitors. Antibodies against LTI29, ERD14, LTI30, and RAB18 were used as described by Nylander et al. (2001). We compared all treatments and patches – eight samples per patch (wild type green patch, wild type white patch, green mutant), in the two growth chambers – normal (20°C/10°C d/n) and cold temperatures (15°C/5°C d/n).

### Leaf Temperature

Measuring leaf temperature is challenging (Vermeulen et al., 2012) and measuring direct temperature differences between intra-leaf patches was beyond our capabilities. Hence, we used an IR camera (T-335, FLIR), at 8 am, to estimate the whole leaf temperature by proximate sensing. Image analysis has been done by FLIR Tools software package. We compared mutant green leaves to wild type variegated leaves as we could not distinguish white and green patches on variegated leaves. We took thermal images of five plants per growth temperature (normal/cold) and plant type (green/white), a total of 20 plants. Photos were taken at 8:00 to resemble natural conditions that expose plants to cold temperatures and low radiation. We assumed that if differences between green and white patches are distinguishable, they are likely to be apparent at this time of the day. Each image was divided to 4–7 rectangle sections, an average, minimum, and maximum temperatures were calculated per section according to pixel coloration, a reflection of the IR radiation. Each section served as a repetition, summarized to approximately 25 repetitions per combination of leaf type (green/varigated) and growth temperature (normal/cold).

### Photosynthetic Activity and Plant Growth

Plants were harvested at February 13th 2013 after 90 days of growth. Shoot dry weight (DW) served as a measure of plant development. Shoots were harvested and dried (65°C, 48 h) before measurement with analytical scale (Entris^®^ Analytical Balance, Sartorius Corp., Goettingen, Germany). Five shoots per plant type and growth chamber were measured. To examine photosynthetic response of white and green patches to thermal conditions – we measured photosynthesis rate in three different temperature conditions. Five individuals per plant type (wild type or green mutant) and growth chamber temperature (normal/cold) were measured in three temperature conditions. The plants were measured in three different rooms to provide the partitioning of air temperatures, finely dictated by infra-red gas analyzer (IRGA) (LI-COR 6400, LI-COR Biosciences, Lincoln, NE, United States). Leaf gas exchange parameters were measured using an (IRGA) coupled with a 2 cm^2^ leaf fluorescence chamber (Li-6400-40 leaf chamber fluorometer; Li-Cor., Inc.) This setup allowed an estimation of the non-photochemical quenching parameter (NPQ). NPQ is a good estimator of the heat dissipation, or the light energy that was not used by the plant for photosynthesis (Murchie and Lawson, 2013) To provide 20°C we used a growth chamber; 15°C was measured in the lab; to measure photosynthesis efficiency in 5°C we used a cold room. *Photosynthetic efficiency* is a measure of the maximal PSII efficiency, expressed by the ratio Fv/Fm (Papageorgiou and Govindjee, 2004). Fm is the maximum fluorescence re-emitted from the measured leaf; Fv is expressed as Fm-Fo, where Fo denotes fluorescence yield in the absence of actinic light (Maxwell and Johnson, 2000). Fv/Fm indicates the efficiency of PSII photochemistry during conversion of excitation energy to chemical energy in the photochemical electron transport chain. In healthy plants, this efficiency is about 80% or 0.8 Fv/Fm; values of Fv/Fm that are lower than ∼0.8 are considered as injurious levels, given that plants are measured at the same conditions (Bjorkman and Demmig, 1987; Johnson et al., 1993). Photochemical efficiency was always measured at the same time of the day for all plants (8:00–10:00 am). We measured darkened leaves with a MINI-PAM (Walz GmbH, Effeltrich, Germany). Leaves were dark-adapted for 20 min to ensure full oxidation of the electron transport chain and disengagement of the qE thermal energy-dependent quenching. We measured four combinations of patches and growth temperature (white and green patches in regular and cold growth temperatures). We used at least four patches for each patch combination as repetitions. We measured net photosynthetic rate by an IRGA, following light accumulation curve procedure as described by Carriquí et al. (2018). Net photosynthesis was measured in CO_2_ concentrations leveled to 400 μmol mol^-1^ (approximating ambient concentration, ∼378 μmol mol^-1^) and under three levels of photosynthetic active radiation (PAR = 100, 500, 1,500 μmol photons m^-2^ s^-1^). The sequence of light curve performed was PAR = 100, 500, 1,500, 100, 1,500, 100 with 3 min intervals. The initial goal of this sequence was to trace light reaction after gradual increase of light intensity, a recovery, and a sharp increase of light intensity. Finally, for simplification, we considered only the first three sequential repetitions of PAR levels (100, 500, and 1,500 μE m^-2^ s^-1^ performed in this order).

## Results

### Morphology and Phytochemistry of White and Green Patches

**Figure 1:**
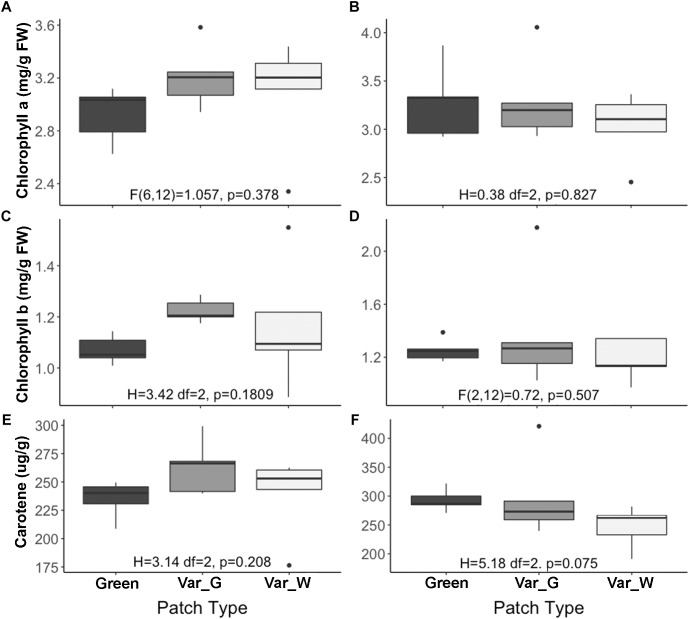
Biochemical content of *Silybum marianum* white and green patches. The *X* axis shows three categories of patch types: Green, patch of a green mutant; Var-G, green patch on a variegated leaf; Var-W, white patch on a variegated leaf. The left figures **(A,C,E)** describe measurements of plants grown under normal conditions (20°/10°C d/n). The right figures **(B,D,F)** describe measurements of plants grown under cold conditions (15°/5°C d/n). **(A,B)** Chlorophyll-*a* content; **(C,D)** Chlorophyll-*b* content; **(E,F)** Total carotene content (mg/g). Results of one-way ANOVA test are given per figure. Boxplots represent median. The lower and upper hinges correspond to the 25 and 75th percentiles. Dots represent outliers.

Our results suggest that variegation in *S. marianum* is created by an intercellular space above the chlorenchyma rather than by pigments (Supplementary Material S2). We found that stomata length in white and green patches did not differ across growth temperature and leaf aspect. Interestingly, compared to green patches – white patches seem to have higher density of stomata at the abaxial side, and lower density on the adaxial side, although we noticed differences with significance of *p* = 0.06 only in the case of plants growing under cold temperatures (Table 1). Biochemistry of the green and white patches did not show any clear and significant differences: total carotene, chlorophyll-*a* and chlorophyll-*b* did not differ significantly (Figure 1). Dehydrin content did not show any significant difference between patches or growth temperatures, and hence is not presented here.

**Table 1:** Stomata density and length of *Silybum marianum* white and green patches.

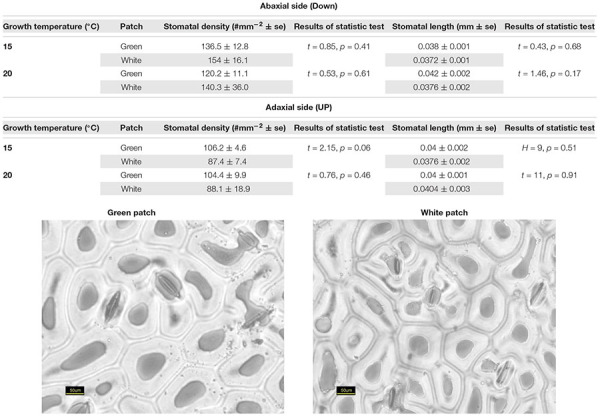

*Microscope images are randomly chosen from each patch and do not represent a typical character of the stomata in the different patches. Taken from transparent imprints, the gray colors of the images have no biological meaning. Pair-wise *t*-test results are given, or Mann-Whitney U in case that data was not distributed normally (*n* = 5)*.

### Optical Attributes of White, Green, and Mixed Patches

**Figure 2:**
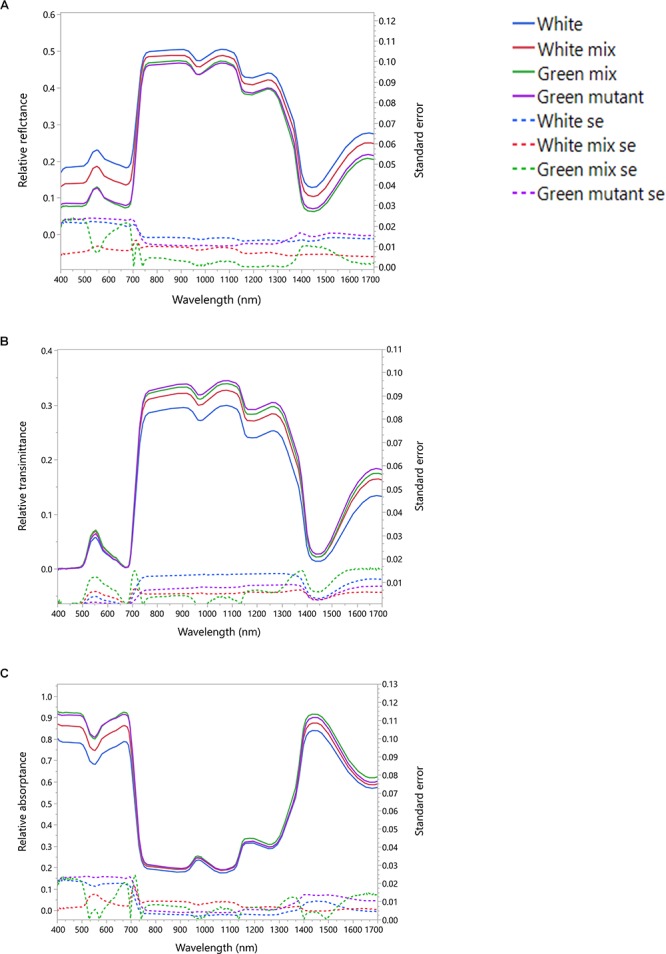
Averaged spectral signatures of *S. marianum*. The spectral signature was measured in four different patch types, along a gradient of white to green variegation. White – is an entirely white patch on a variegated wild type leaf. Green mutant – is an entirely green patch, on a green leaf mutant. White mix and Green mix – are the intermediate patches on a wild type variegated leaf, where the white mix is a patch dominated by white variegation, and the green mix is dominantly green. **(A)** Leaf averaged reflectance and standard error; **(B)** leaf averaged transmittance and standard error; **(C)** leaf averaged absorptance and standard error. Dotted lines represent standard error (se).

The measurements obtained by the integrated sphere provided information on the leaf reflectance (Figure 2A), transmittance (Figure 2B) and absorptance (Figure 2C). The averaged reflectance of the white patches was with the highest intensity for all the spectral range. The white dominated mixed patches intensity was intermediate and the green and green dominated mixed patches had the lowest intensity. Therefore, less VIS radiation is available for the chlorophyll in the white patches. The measured transmittance showed very similar intensities in the VIS region for all patches types. Thus, the absorptance of VIS radiation in the white patches is smaller than the other patches (Figure 2C). The radiation absorptance in the NIR had no visible differences between all four patches as a result of the opposite reflectance and transmittance trends in that spectral region. In the SWIR spectral region the white patches reflected more than other patches, transmitted less than others and absorbed less than other (Figure 2).

### Thermal Traits of White and Green Patches

**Figure 3:**
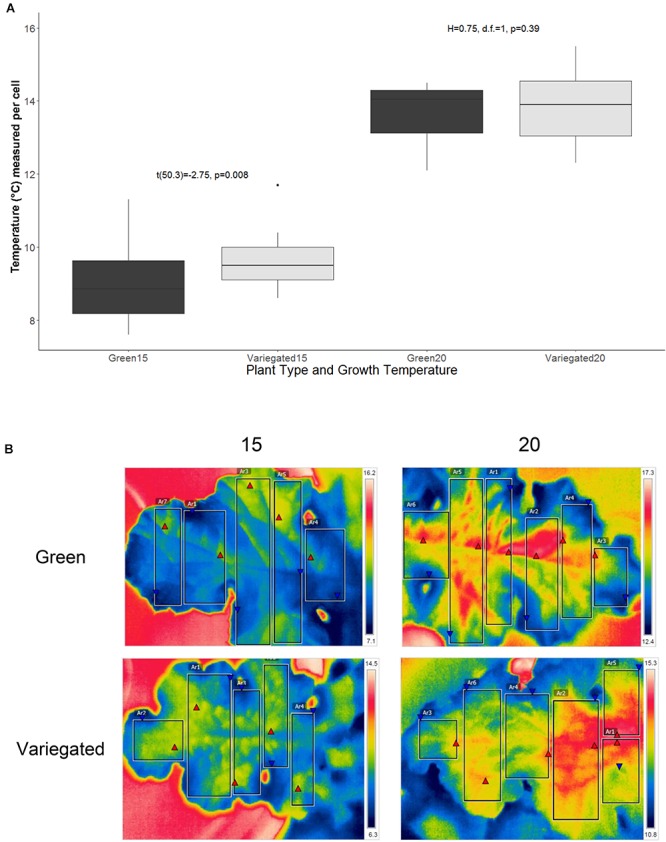
Thermal traits of *Silybum marianum*’s white and green patches. The temperature of five adult leaves per plant type and growth temperature was measured by an IR camera at 8 am. Variegated leaves are the wild-type, green leaves are of the all-green mutants without any white patches. Growth temperature relate to the conditions maintained within the growth chambers. 20 represents the normal conditions (20°/10°C d/n) whereas 15 denotes cold temperatures (15°/5°C d/n). **(A)** Average pixel temperature of variegated and green leaves was calculated from 4 to 6 rectangular areas in the leaf image. Results of *t*-test are given within the figure. Boxplots represent median. The lower and upper hinges correspond to the 25 and 75th percentiles. Dots represent outliers; **(B)** Four typical IR images that served as the data to analyze thermal traits. Each leaf was divided to 4–6 rectangular areas, from which various values are presented and calculated. These include min (red triangular) and max (blue triangular) temperatures, and average pixel temperature, with color-temperature scale on the right side of the image.

Figure 3 presents the thermal difference between wild type and all-green mutant leaves of *S. marianum*. Plants grown in mildly cold temperatures (15°C/5°C d/n) showed that wild type leaves were ∼7% warmer (9.5 ± 0.15°C average ± se) than all-green mutant leaves (8.9 ± se 0.17°C average ± se). Overall higher temperatures (13.8°C) were measured on leaves growing in the normal temperatures (20°C/10°C d/n). However, our measurements did not detect significant difference between patch types of plants grown in normal temperatures (Figure 3A). Figure 3B demonstrates the apparent difference between the two leaf types in two temperature regimes, in images taken by IR camera.

### Photosynthetic Activity and Efficiency of White and Green Patches

**Figure 4:**
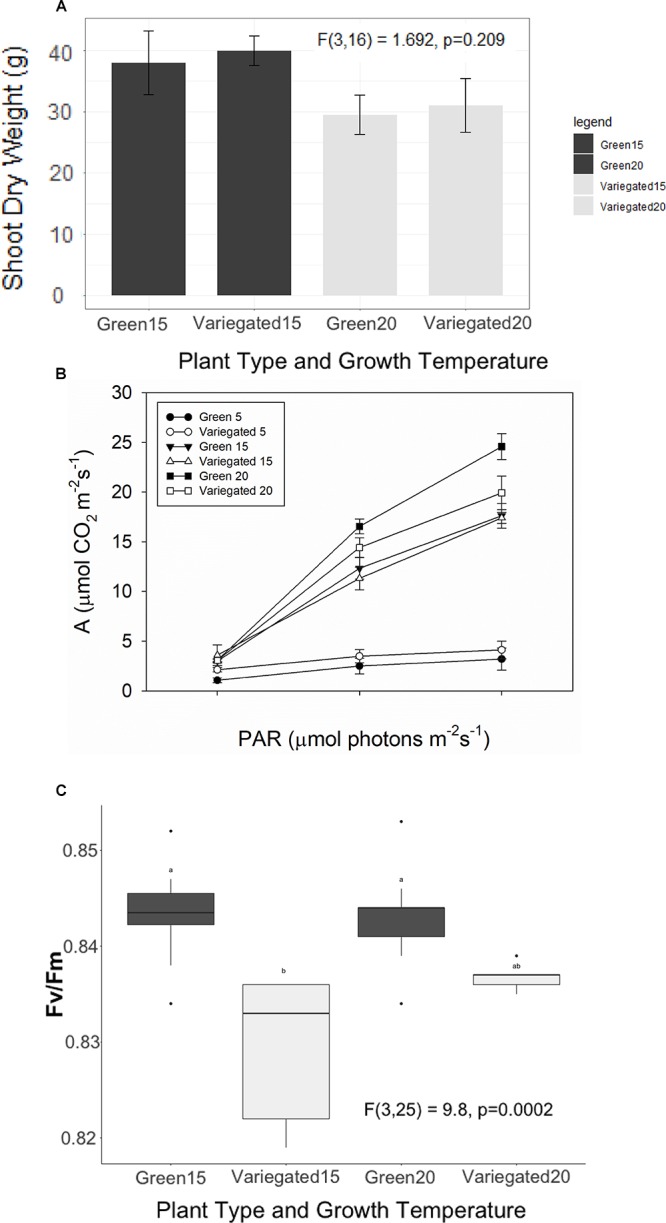
Photosynthetic efficiency of *Silybum marianum* white and green patches. Variegated denotes wild type patched leaves, Green represents an all-green mutant. **(A)** Plant growth. The values represent the total shoot dry biomass, as harvested at the end of the experiment; **(B)** Photosynthetic efficiency as measured by Infra-Red Gas Analyzer (LICOR-6400) for plants grown at normal winter temperatures (20°/10°C d/n). Light curves were measured at ambient CO_2_ level (380 ppm) following a short time acclimation (1 h) to 5°, 15°, or 20°C; **(C)** Maximal PSII efficiency (Fv/Fm) measured by WALTZ miniPAM after a dark period. Boxplots represent median. The lower and upper hinges correspond to the 25 and 75th percentiles. Dots represent outliers. Different letters denote significant differences.

All-green mutants appeared to grow 28% heavier shoots than wild type plants (38.9 ± 2.7 g/30.2 ± 2.6 g average ± se). However, these results are not significant statistically. In colder temperatures, plants appear to grow less, but again, the differences between the wild-type and the green mutant are not significant (Figure 4A). We found a similar result for the NPQ – no significant differences between the patch types. Interestingly, the green patches appear to have higher NPQ in all conditions, meaning white patches may be more efficient, or dissipating less light for heat. However, the differences were not significant and are not presented in figures. The photosynthetic efficiency dropped as *S. marianum* plants were exposed temporarily to colder temperatures (Figure 4B), with the least photosynthesis rate at exposure to 5°C. Looking to compare white and green patches – our measurements did not find any significant advantage to one patch over the other, in plants grown at normal temperatures (Figure 4B). Similar insignificant differences between the two patches were found in plants grown in colder temperatures (not presented). The maximal PSII efficiency (Fv/Fm) was significantly lower in white patches that grew in normal temperature conditions (Figure 4C). However, the values of all four patches and growth temperatures were high and therefore there is no biological means for these minor differences: all plants presented values of considerable high levels of photosynthetic activity. Efficiency of 80% is considered as the threshold for healthy unstressed plants (Fv/Fm > 0.8).

## Discussion

We studied white and green patches in *S. marianum*, aiming at figuring out if there are any distinguishable physiological differences between them. We tested the hypothesis that white patches provide a physiological advantage to the leaf, as they can alleviate temperatures during cold winter days, allowing improved thermal conditions for more efficient photosynthesis. In accordance to our predictions, we did not find significant differences in some phytochemical features of the two patch types. We found that the white colored patch is a result of a structural difference (sub-epidermal air spaces), and that the stomata dispersion did not differ significantly between green vs. white patches (*p* > 0.05). Under low temperature conditions, we measured higher temperatures on wild-type plants, in comparison to all-green mutant plants. We did not find any significant difference in the photosynthetic activity and NPQ of the two patches, suggesting that white patches are not hindering photochemical efficiency.

### White and Green Patches Are Distinguished Anatomically and Morphologically and Not Biochemically

Our study confirmed that the white variegation in *S. marianum* is structural: sub-epidermal air spaces, is the mechanism creating the patches of white coloration (Supplementary Material S2). The optical features that we measured are in accordance to this finding (Figure 2). In the VIS spectral range, white patches exhibited the lowest absorptance rates, due to high reflectance, meaning that the morphological coloration is correlated with lower absorptance rates. These findings suggest that pigment functioning is reduced in the white patches as the pigment levels are similar to those in the green patches, but in the white patches, the miniature air spaces are masking the chlorophyll. Regarding water content, affecting the SWIR spectral range, although in the water-affected region, the relatively high reflectance might be partly increased by the air spaces (Karnieli et al., 2013), which is a leaf structure property. The absorptance in the SWIR region is the lowest for the white patches as they might have higher water content than green patches. Since there is no information about the actual water content, the above mentioned difference can only be assumed. Sheue et al. (2012) explained how the epidermal tissue, when loosely connected to the mesophyll, is creating tiny air spaces that appear as light areas in *Begonia*. According to Sheue et al. (2012) the air spaces are photosynthetically active. Our results support the findings and interpretation of Sheue et al. (2012). Nevertheless, we noticed some slight differences in stomata density (Table 1), where white patches seem to have in average more stomata at the abaxial side as compared to the adaxial side. These differences were not significant statistically (*p* > 0.05). Having more stomata on the leaf’s lower surface (hypostomy) is common (Fahn, 1990), mainly in fast growing plants, such as annuals (Muir, 2015). If this hypostomy is pronounced more prominently in white patches, it may induce different functioning of the photosynthesis dynamics in the two patch types. The stomatal ratio between abaxial and adaxial sides has a role in transpiration and therefore affecting the heat balance of the leaves (Lee, 2018). To show the impacts of distinguished stomatal ratio in white versus green patches, it requires firmer results, and statistics that are more robust. Muir (2015) suggested that stomata ratio is also related to defense against pathogens – more stomata on the adaxial (upper) side means more efficient photosynthesis, but this trend is constrained by a higher risk of pathogen intrusion. We did not look at stomata resistance that can provide the plant with the flexibility to respond to a variety of stresses (Lu, 1989). Nevertheless, a variety of stomata spacing, ratio and resistance, may hypothetically enable the leaf a greater flexibility of response arsenal to different environmental conditions. Hence, if we can show that such a difference between stomata density in the two patch type exists, it may suggest that white variegation is related to a higher level of physiological flexibility. At this point, this is only hypothetical explaining variable, as it is uncertain if stomatal resistance would show any differences between the patch types.

### White Patches Can Provide Thermal Advantages

We found that in plants grown in a cold temperature regime (15°C/5°C d/n), the wild type leaves were significantly warmer than those of all-green mutants (Figure 3). The major factors affecting the energy budget of a leaf are energy gain or loss by absorptance/emission of long and short-wave radiation, energy gain or loss by convection and energy loss by transpiration. The temperature of the leaf relative to the air temperature will vary depending on external parameters including light intensity, temperature, wind and relative humidity. Hypothetically, factors that may cause slightly higher temperatures in the white patches are: (1) absorptance. However, lower absorptance of visible light, as appeared in the white patches is likely to result in less heat gain by radiation. Hence, absorptance is not the leading factor to warmer white patches; (2) active stomata structure – more stomata means more efficient photosynthesis, that may affect the heat balance. However, our results did not confirm significant differences in stomata structure. (3) Sub-epidermal air spaces alleviate the temperatures locally. This effect may be the result of reduced heat convection, or another mechanism. In other words, the results support the hypothesis that white variegation in *S. marianum* are able to increase leaf temperatures presumably functioning as miniature greenhouses. The patchy nature of leaf variegation in *S. marianum* can provide the plant with near optimal thermal micro-conditions in a variety of climatic conditions that are typical to the Mediterranean winter. Leaf thermoregulation is a key factor in carbon economics, among other parts of leaf optimization to maximize carbon gain in different and dynamic environments (Michaletz et al., 2016), and probably concerning other metabolic functions. In addition, the structure and distribution of leaf veins play a key role in mitigation of frost stress (Stevenson, 2015; Lim et al., 2017) or improved transport in the phloem channels. Hypothetically, regulating the temperature in the veins by sufficient structure can reduce the exposure to freezing point by shortening the time of exposure, and the range of temperatures. This hypothesis has to be studied in more details to get further support.

### Photosynthetic Activity in White Patches Is as Efficient as in Green Patches

Our results did not support the hypothesis that white patches alleviate photosynthetic rate by creating warmer spots under cold air temperature conditions. However, the results do suggest that photosynthesis in white patches is not inferior to green patches: in spite of the apparent screening of chlorophyll in white patches, the photosynthetic efficiency and activity are the same as in green patches, in all tested conditions (Figure 4). The shoot dry weight of the all-green mutant seems to be marginally heavier (Figure 4A), though insignificantly. Apparently, all-green mutants are growing more efficiently in controlled temperatures. Nevertheless – they do not prevail in natural conditions, being a rare phenotype in a dominant population of white variegated wild type (Lev-Yadun, 2017). The dominance of the white variegated phenotype shows that the advantage of white patches is overtaking the potential disadvantages of partly masking the direct green tissue. In other words, even though visible light absorptance rates in the white patches are reduced – the same chlorophyll content is more effective under higher temperature conditions in a warmer microenvironment. Our results suggest that the thermoregulation flexibility of the white patches may be the mechanism behind the dominancy of white patches in *S*. *marianum* and maybe in other species. If indeed as suggested, the white patches also serve as visual defense from herbivory (Lev-Yadun, 2003, 2016) – the gain from variegation is even larger.

## Conclusion

This study showed that white variegation on *S*. *marianum* leaves, the outcome of sub-epidermal air spaces, may provide the leaf with thermal advantages during cold winter days. These findings can give an additional explanation to patterns of colors in variegated plant leaves, revealing a physiological mechanism in the evolution of non-green coloration of leaves, and of its physiological function in leaves.

## Author Contributions

OS lead the experiments and wrote the process. LS was in charge of plant growth and conducting all experiments. SL-Y provided seeds of WT and green mutants and initiated ideas. RS and IH analyzed the hyperspectral data. SV-C assisted with data analysis and presentation. SR coordinated the study. All authors contributed to writing the manuscript.

## Conflict of Interest Statement

The authors declare that the research was conducted in the absence of any commercial or financial relationships that could be construed as a potential conflict of interest.
