# Supplementary material for: Thermal Benefits From White Variegation of Silybum marianum Leaves
Source: Front Plant Sci. 2019 May 24;10:688. doi: 10.3389/fpls.2019.00688 (PMC6543541; doi:10.3389/fpls.2019.00688)
Supplement: Supplementary file 1 [file Data_Sheet_1.docx]

SM1

| *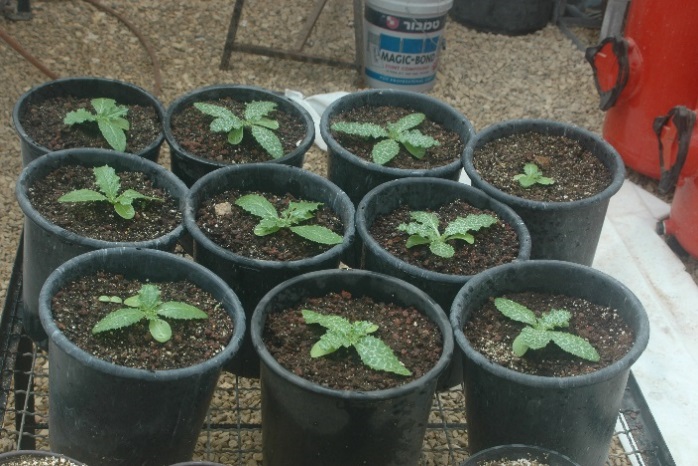*  A | *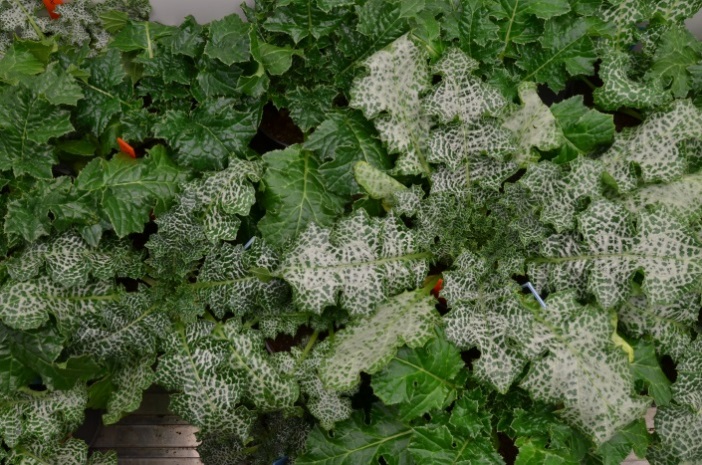*  B |
| --- | --- |
| *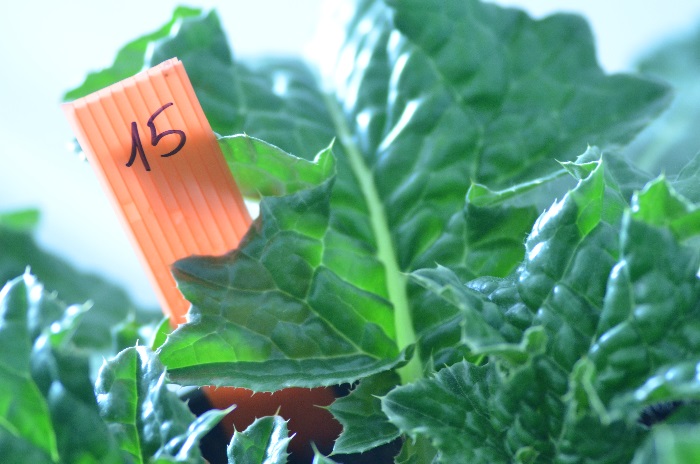*  C | *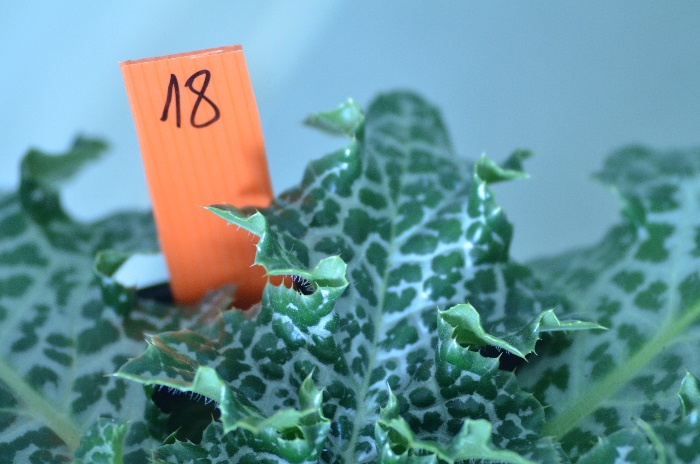*  D |

SM1. Photos of *Silybum marianum* wild type and all-green mutants. All photos taken by Oren Shelef. A) Seedlings at the greenhouse 10 days from sowing, variegated wild type and an all-green mutant are seen; B) Mature plants in a growth chamber, mixed sample of variegated wild type and green mutant; C) An all-green mutant plant at the time when experiments and measurements were performed; D) A variegated leaf of mature wild type plant.

SM2

| *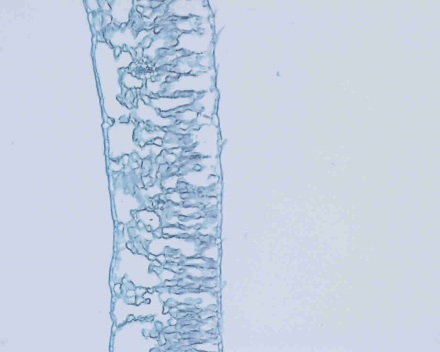*  **100 μm**  A  Abaxial  Adaxial | *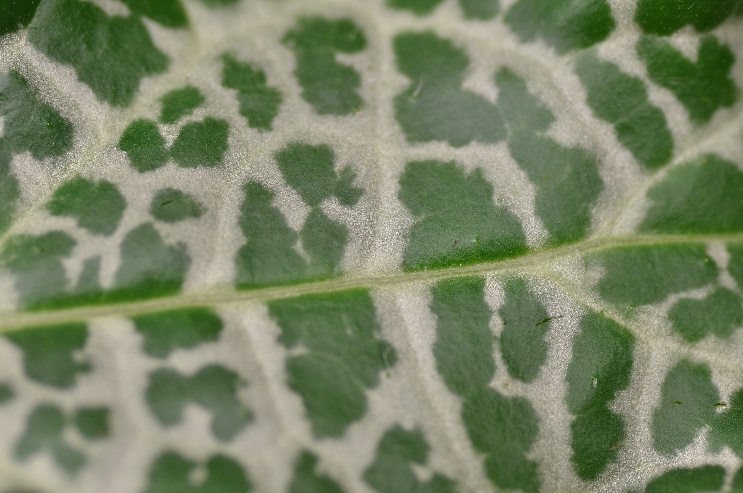*  **1 cm**  B |
| --- | --- |
| *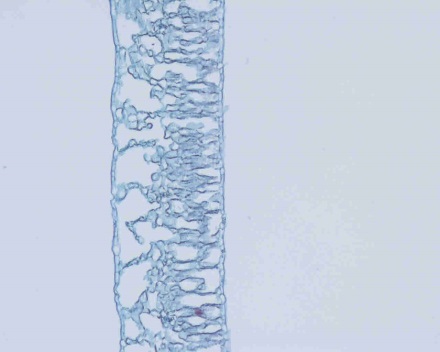*  C  Abaxial  Adaxial | *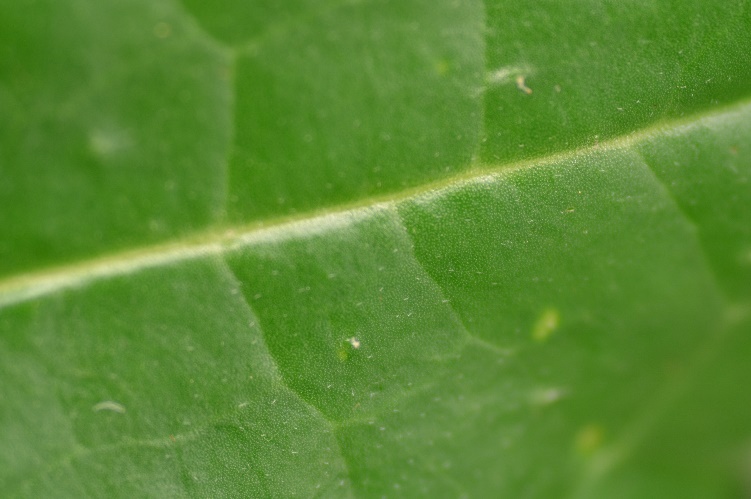*  D |

SM2. Morphological and anatomical structure of *Silybum marianum* white and green patches. A) Black arrows mark the large air spaces of a white patch in the left side of the figure. These air spaces are the typical structure of white patches; B) Wild type leaf of *S. marianum*. White patches are clearly covering a large portion of the leaf area; C) green palisade cells of the adaxial (upper) side of the leaf are abutted to the epidermis and only small air spaces can be found under the stomata; D) Green mutant leaf of *S. marianum*. Large air spaces and white variegation are absent.
